# Influence of temperature gradient of slab track on the dynamic responses of the train-CRTS III slab track on subgrade nonlinear coupled system

**DOI:** 10.1038/s41598-022-18898-y

**Published:** 2022-08-27

**Authors:** Qingyuan Xu, Shengwei Sun, Yi Xu, Changlin Hu, Wei Chen, Lei Xu

**Affiliations:** Department of Civil Engineering, Central South University, Changsha, 410075 China

**Keywords:** Engineering, Civil engineering

## Abstract

Temperature is an important load for ballastless track. However, there is little research on the system dynamic responses when a train travels on a ballastless track under the temperature gradient of ballastless track. Considering the moving train, temperature gradient of slab track, gravity of slab track, and the contact nonlinearity between interfaces of slab track, a dynamic model for a high-speed train runs along the CRTS III slab track on subgrade is developed by a nonlinear coupled way in ANSYS. The system dynamic responses under the temperature gradient of slab track with different amplitudes are theoretically investigated with the model. The results show that: (1) The proportions of the initial force and stress caused by the temperature gradient of slab track are different for different calculation items. The initial fastener tension force and positive slab bending stress have large proportions exceeding 50%. (2) The maximum dynamic responses for slab track are not uniform along the track. The maximum slab bending stress, slab acceleration, concrete base acceleration appear in the slab middle, at the slab end, and at the concrete base end, respectively. (3) The maximum accelerations of track components appear when the fifth or sixth wheel passes the measuring point, and at least two cars should be used. (4) The temperature gradient of slab track has a small influence on the car body acceleration. However, the influences on the slab acceleration, concrete base acceleration, fastener tension force are large, and the influence on the slab bending stress is huge.

## Introduction

Ballastless track is a modern technology in railway. Compared with the ballasted track, the ballastless track has significant advantages, such as better stiffness uniformity, excellent integrity, lower maintenance cost, higher running stability, and better durability. With the economic and technological development, it has been extensively used worldwide^1^.

There are four main types of ballastless track used in China^2^: the China Railway Track System (CRTS) I, II, and III slab track, and the double block (DB) ballastless track. The cement asphalt (CA) mortars^3^ in CRTS I and II slab track, which are easily damaged, are replaced by the self-compacting concrete in CRTS III slab track^4^ to reduce the maintenance work effectively. Since 2010, many high-speed railways in China have adopted the CRTS III slab track due to its high performance.

**Figure 1:**
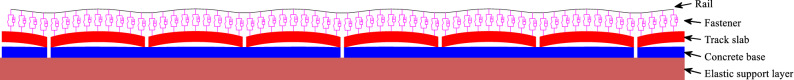
The schematic of initial deformation and gap caused by the temperature gradient of ballastless track.

Besides its advantages, the ballastless track has its limitations. One limitation is the influence of the temperature load. Figure 1 shows the schematic of initial deformation and gap caused by the temperature gradient of ballastless track. The deformed track and gap negatively influence the system dynamic responses and should be studied in detail.

Temperature load is a vital load type for the ballastless track. Many studies have been made on the temperature distribution in the ballastless track. Ou and Li^5^ predicted the temperature field in CRTS II slab track using a one-dimensional analytical solution. Yang et al.^6^ established a three-dimensional (3D) temperature field model of DB ballastless track considering the geographical location and environmental conditions for calculating the track temperature distribution based on the climatic data. Lou et al.^7^ presented a temperature action model suitable for DB ballastless track and bridge structure to study the distribution regularities of temperature spectra and the relationship between the atmospheric temperature and structure. Liu et al.^8^ studied the temperature distribution and its influence factors in the asphalt supporting layer of CRTS III slab track by combining numerical simulation and field measurement. Zhao et al.^9^ constructed a scaled specimen with CRTS II slab track on a girder bridge in a laboratory to study the temperature distribution laws. Using phase change material, Jiang et al.^10^ designed a novel coating in CRTS II slab track to reduce the slab arching caused by the continuous high temperature by optimizing the insulation effects of the coating with different modules.

Under the temperature of ballastless track, the deformations are generated in the ballastless track, and some scholars conducted researches on these aspects. Using the energy method, Ren et al.^11^ deduced an analytical expression for CRTS II slab track to investigate the slab upwarping deformation due to the rising temperature. Chen et al.^12^ researched the stability of CRTS II slab track caused by the initial upwarping deformation at high temperatures with the finite element method, as well as the slab warping deformation due to the non-uniform temperature field with the analytical method^13^. Cai et al.^14^ established a 3D finite element model (FEM) of the CRTS II slab track considering the joint damage to explore the arching mechanism of joints.

The temperature load also greatly affects the mechanical and damage properties of ballastless track. Many kinds of related research have been conducted. Cho et al.^15^ investigated the effect of steel ratio on the stress and crack width of DB ballastless track under temperature load using ABAQUS software. Liu et al.^16^ studied the damage mechanism and development of the slab joint under temperature rise for CRTS II slab track using the damaged plasticity model and cohesive zone model respectively for the concrete and interface. Li et al.^17^ explored the interface damage of CRTS II slab track under temperature load with a 3D FEM using the cohesive zone element to model the interface. And recently, the interfacial failure and arching of the track with reinforcement bars due to thermal effects were further studied^18^. Xu et al.^19^ studied the carrying-out debonding repairment on the mechanical characteristic as well as the interface damage of CRTS II slab track under slab temperature gradient. Cui et al.^20^ researched the interface damage of CRTS II slab track with different damage levels of joint concrete under the temperature loads.

The temperature loads in Refs.^15–20^ cannot reflect the time-varying characteristic of the actual temperature loads. Some scholars adopted the measured or calculated time-varying temperature loads to research ballastless track’s mechanical performance and damage. Zhong et al.^21,22^ imported the measured time-varying temperature loads to a 3D FEM of CRTS II slab track to investigate the curling behaviors as well as the interface stresses in the construction stage due to the daily changing temperature. Zhu et al.^23^ studied the mechanical characteristic and interface damage evolution of DB ballastless track under the cyclic time-varying slab temperature gradient load based on the meteorological data of Guangzhou district in China in the year 2001. Song et al.^24^ researched the temperature field, temperature deformation, and interfacial damage for the CRTS II slab track in Nanjing, China.

Besides the temperature load, the train load is another important load for railway track. Some scholars researched ballastless track’s mechanical characteristics and damages considering both the train and temperature loads. Using a 3D FEM, Xu and Li^25^ investigated the stress in CRTS I slab track under combined loads. Ren et al.^26^ researched the influence of CA mortar damage on the carrying capacity of CA mortar and track slab for CRTS I slab track under the joint action of slab temperature gradient and train load, and proposed the repairing criteria for CA mortar. Zhang et al.^27^ investigated the interlayer debonding of CRTS II slab track based on viscoelastic theory under temperature and vehicle loads. Wang et al.^28^ researched the vertical deformation and CA mortar stress of CRTS II slab track under 5 different temperature loads and 4 temperature and train load combinations. Li et al. ^29^ established a 3D FEM to investigate the joint damage laws of CRTS II slab track under temperature and vehicle loads. Zhu et al.^30^ studied the interface damage evolution of CRTS II slab track under the joint action of vehicle dynamic load and temperature change, as well as its influences on the track dynamics. Using the track irregularities caused by the temperature load as an excitation, a coupled dynamic analysis was conducted for CRTS II slab track due to slab temperature deformation^31^.

At present, many studies have been conducted on the temperature distributions of ballastless track, and the deformation, mechanical, and damage characteristics of ballastless track, which were caused by the temperature load of ballastless track or by the combined train and temperature loads of ballastless track. However, most of these studies focused on CRTS I^25,26^, CRTS II^5,9–14,16–22,24,27–31^ slab track, and DB ballastless track^6,7,15,23^. While few studies focused on CRTS III slab track^8^. Moreover, most of the mechanical models used in these studies are static, and few studies^30,31^ focused on the system dynamics under the joint action of temperature load of ballastless track and train load. Thirdly, the related coupled dynamic models in Refs.^30,31^ do not consider the nonlinear interface contact in ballastless track, and the dynamic clapping action at the interfaces due to the interlayer gaps cannot be well simulated. The conclusions in Ref.^32^ show that the gaps between the interlayers of ballastless track significantly influence the system dynamics and should be considered.

Based on the findings of train/vehicle-ballastless track coupled dynamics^30–36^, considering the train load, gravity load of slab track, temperature gradient load of slab track, and contact nonlinearity of slab track, a high-speed train-CRTS III slab track on subgrade coupled dynamic model is established. The initial track states and system dynamics under different temperature gradient loads of slab track are studied and analyzed in depth.

## Coupled dynamic model

The calculation results of the train and track calculated by a 3D train-track-subgrade nonlinear coupled dynamic model with the track and subgrade modeled by 3D solid elements are more precise than those calculated by a 2D train-track on subgrade nonlinear coupled dynamic model using the spring to model the elastic support layer of track to reflect the elastic deformation of the subgrade. However, considering the small time step, large load steps, small mesh size, huge degrees of freedom (DOFs), and nonlinear contact are needed in the simulation, the calculations will be extremely time-consuming. The detailed explanations can be found in Ref.^37^. Moreover, Nguyen et al.^38^ compared the dynamic responses of a simplified 2D and full 3D high-speed vehicle-ballasted track on subgrade coupled system, respectively. It concluded that the 2D model using the spring to model the subgrade can meet the precise requirement of the practical engineering and can be used to predict the train and track dynamic responses. Zhai and Cai^39^ verified the 2D train-track on subgrade nonlinear coupled dynamic model using the spring to model the elastic support layer of track with the field experiments, the calculated results are close to the measured results. Thus, a simplified 2D train-CRTS III slab track on subgrade coupled dynamic model is adopted in this study.

**Figure 2:**
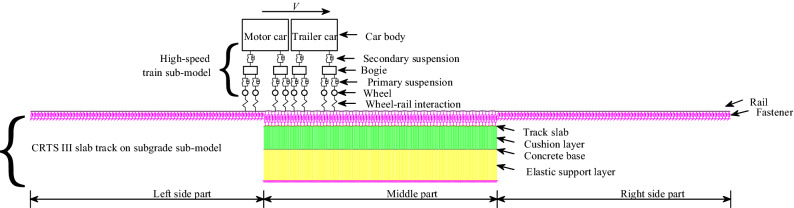
The schematic of the coupled dynamic model.

Figure 2 shows the schematic of the coupled dynamic model developed using ANSYS parametric design language. In the model, a high-speed train moves forward along the CRTS III slab track on subgrade at a constant speed *V.*

The model includes three parts. The wheel-rail interaction submodel based on Hertzian nonlinear contact theory is the same as those in Refs.^39–41^. The details of the other two submodels are as follows.

### High-speed train submodel

A typical high-speed train has 4 motor and 4 trailer cars. However, according to the research in Ref. ^33^, the dynamic responses with 2 cars are almost identical to those with 8 cars. Thus, 2 cars are used in the submodel to improve the computation efficiency of the coupled system.

In Fig. 2, the primary and secondary suspensions are used to connect the bogie and wheel, and the car body and bogie, respectively. The suspensions are simulated by the spring-damper elements. The wheel can only move in vertical motion, while the bogie and car body have two DOFs: pitch and vertical motions. For each car, the DOFs are 10, and the total DOFs for a train with 2 cars are 20. The detailed dynamic equations for a car could be consulted in Refs.^39,40^. The dynamic parameters of the train are given in Table 1.

**Table 1:** The dynamic train parameters.

Component	Item	Unit	Values	Component	Item	Unit	Values
			(Motor car/Trailer car)				(Motor car/Trailer car)
Car body	Mass	kg	21,200/22,000	Primary suspension	Stiffness	kN/m	1040/700
Bogie	Mass	kg	1700/850	Primary suspension	Damping	kN.s/m	35/15
Wheel	Mass	kg	1100/950	Secondary suspension	Stiffness	kN/m	400/350
Car body	Pitch inertia	kg.m^2^	1,370,000/1,370,000	Secondary suspension	Damping	kN.s/m	35/60
Bogie	Pitch inertia	kg.m^2^	3600/850				

At the beginning time, the first wheel of the train is located 36.855 m behind the middle of the model. The train moves forward 106.785 m at 300 km/h. The simulation time step is 0.0001 s to reasonably consider the high-frequency dynamic responses.

### CRTS III slab track on subgrade submodel

The submodel uses multi-scale modeling technology, as shown in Fig. 2. It consists of three parts. Only fastener and rail are used in the two side parts. While a refined model with a small mesh size is used in the middle part. The lengths of each side and middle parts are 255.465 and 136.08 m, respectively. The total model length is 647.01 m.

**Figure 3:**
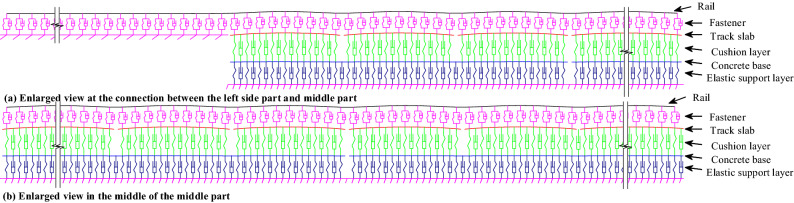
Enlarged view of the submodel.

The enlarged views of the submodel around the connection between the middle and left side parts, as well as in the middle of the middle part are shown in Fig. 3a and b, respectively.

In the middle part, 24 slabs with 5.67 m length and 8 concrete bases with 17.01 m length are modeled, respectively. Small mesh whose length is the 1/6 fastener spacing is adopted to reasonably reflect the complex contact relation between different track components.

Beam elements are employed to model the concrete base, slab, and rail. Spring-damper elements are employed to model the fastener. Contact elements that consider only the compressive force are employed to model the interface between slab and concrete base and the elastic support of the subgrade to reflect the complex time-varying dynamic interlayer contact relation under the combined loads. The submodel parameters are given in Table 2.

**Table 2:** The parameters for CRTS III slab track on subgrade submodel.

Component	Item	Unit	Values	Component	Item	Unit	Values
Rail	Elastic modulus	Pa	2.1 × 10^11^	Interface between slab and concrete base	Contact stiffness	N/m	1.31 × 10^8^
	Density	kg/m^3^	7800		Contact damping	kN.s/m	1.16 × 10^4^
	Area	m^2^	77.45 × 10^–4^	Elastic support layer	Contact stiffness	N/m	3.10 × 10^7^
	Moment of inertia	m^4^	3.217 × 10^–5^		Contact damping	kN.s/m	1.36 × 10^4^
Fastener	Stiffness	kN/mm	40	Slab	Elastic modulus	Pa	3.6 × 10^10^
	Damping	kN.s/m	20	Concrete base	Elastic modulus	Pa	3.25 × 10^10^
	Space	m	0.63	Slab and concrete base	Density	kg/m^3^	2500

### Solution and post-processor of the coupled dynamic model

The initial states greatly influence the dynamic characteristics of the coupled system. Therefore, a static analysis is firstly performed under the joint action of train gravity, track temperature gradient, and track gravity. Then, the dynamic simulation for a train moving forward will be performed, taking the static analysis results as the initial conditions. More details on the solution process can be consulted in Refs.^42,43^.

The total node and element numbers in the coupled dynamic model are 5759 and 8291, respectively. The simulation steps for each load case are 11,604. Therefore, it is impossible to store all time histories in the result file due to the limited hard disk volume in an ordinary personal computer. It is vital to choose reasonable post-process output parameters to obtain a balance between the result file size and the accuracy of maximum dynamic responses. In this study, the nodes and elements in the middle of two adjacent fasteners and at the fastener position from the 9th to 15th slab are chosen as the output range.

## Numerical simulation

The dynamic responses of the coupled system have close relation with their initial states. In this Section, the initial states are studied first. Then, considering the influence of the initial states, the envelope curves of slab track as well as the dynamic responses of the coupled system for typical load cases are studied. Finally, the maximum dynamic responses under 7 types of temperature gradient of slab track, whose values *f* are − 45, − 22.5, 0, 22.5, 45, 67.5, 90 °C/m, are investigated and compared. The range of the temperature gradient of slab track is based on the code QCR_9130-2018 in Ref.^44^.

### Initial states of slab track

**Figure 4:**
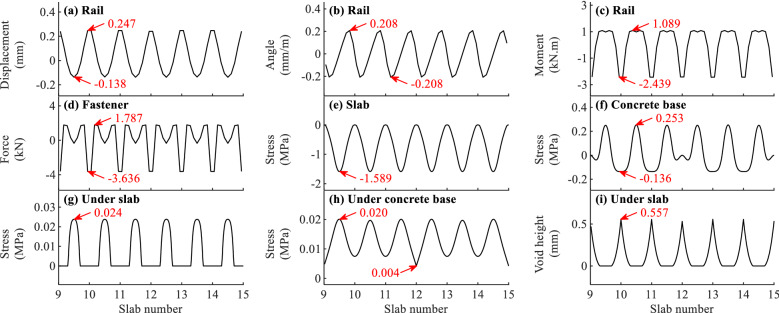
The initial state of slab track for different items when *f* = -45 °C/m.

**Figure 5:**
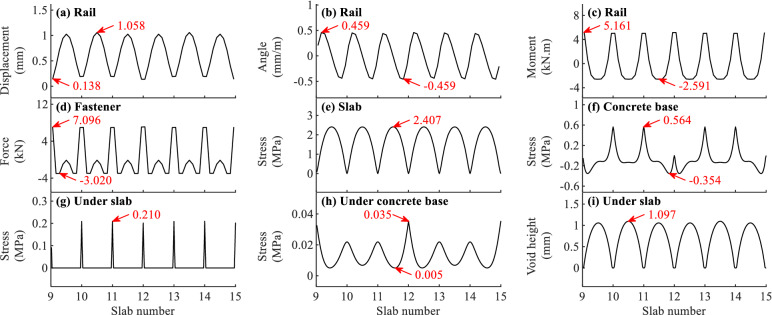
The initial state of slab track for different items when *f* = 90 °C/m.

Different items of initial states of slab track for typical load cases when *f* = − 45 and 90 °C/m are shown in Figs. 4a–i and Fig. 5a–i, respectively.

It can be concluded from Figs. 4–5 that the maximum initial values appear at different positions for different items, and the maximum initial values when *f* = − 45 °C/m differ greatly from those when *f* = 90 °C/m.

As can be observed in Figs. 4a–b and 5a–b that the distributions of rail displacement and rotation angle are different. The maximum rail displacement appears at the end or in the middle of slab. However, the maximum rail rotation angle appears near the end of slab. The initial deformations of rail in Fig. 5a–b when *f* = 90 °C/m are much larger than those in Fig. 4a–b when *f* = − 45 °C/m. For example, the maximum initial displacement of rail when *f* = 90 °C/m is 1.058 mm, which is about 3.3 times larger than that when *f* = − 45 °C/m. The deformed rail when *f* = 90 °C/m will have a more unfavourable effect on the safety and comfort of the running train.

As shown in Fig. 4c, the maximum initial positive and negative rail bending moments appear respectively in the middle and at the end of slab when *f* = − 45 °C/m. Contrarily, they appear at the end and in the middle of slab, respectively when *f* = 90 °C/m, as shown in Fig. 5c. It can also be concluded from Figs. 4c and 5c that the initial rail bending moment has some relation with the temperature gradient of slab track. However, the rail stress calculated with the maximum initial rail bending moment 5.161 kN.m is about 15.2 MPa, which is far less than the allowable rail stress 350 MPa^45^.

Figures 4d and 5d show the initial fastener forces due to the temperature gradient of slab track. One can find that the maximum initial fastener forces appear at or near the slab end. The initial fastener tension force in Fig. 5d when *f* = 90 °C/m is 7.096 kN, which is about 3 times larger than that in Fig. 4d when *f* = − 45 °C/m. The maximum initial fastener tension force when *f* = 90 °C/m is about 40% of the allowable tension force 18 kN for WJ-8 fastener system^46^ and should be taken into consideration in practical engineering.

As shown in Figs. 4e and 5e, the maximum initial negative and positive slab bending stresses occur in the middle of slab. It can also be concluded from Figs. 4e and 5e that the initial slab bending stress in Fig. 5e when *f* = 90 °C/m is much larger than that in Fig. 4e when *f* = − 45 °C/m. And the maximum initial slab bending stress when *f* = 90 °C/m is 2.407 MPa, which is about 85% and close to the allowable concrete tension stress 2.85 MPa for C60 grade concrete in the design code^47^. The slab stress due to the temperature gradient load is large and important for the slab track design and should be considered seriously.

It can be found from Fig. 4f when *f* = − 45 °C/m and Fig. 5f when *f* = 90 °C/m that the maximum initial concrete base bending stresses occur in the middle of concrete base and at the slab end in the middle of concrete base, respectively. As can be observed in Figs. 4f and 5f, the maximum initial concrete base bending stresses are 0.253 and 0.564 MPa, respectively, and the stresses are far less than the stresses in Fig. 4e and 5e for the slab.

Figures 4g–h and 5g–h illustrate the initial contact stresses under the slab and concrete base. One can find that the initial contact stresses are not uniformly distributed. The contact stresses in Fig. 4g–h when *f* = − 45 °C/m and Fig. 5g–h when *f* = 90 °C/m concentrate in the middle and at the end of slab with a large value, respectively. Comparing Fig. 5g with Fig. 4g, the maximum initial contact stress under the slab when *f* = 90 °C/m is 0.21 MPa, whose value is about 7.8 times larger than that when *f* = − 45 °C/m. It can also be deduced from Figs. 4g and 5g according to the initial stress is zero that there are initial gaps at the end of slab when *f* = − 45 °C/m and in the middle of slab when *f* = 90 °C/m.

As shown in Figs. 4i and 5i, the initial gaps distribute at the slab end when *f* = − 45 °C/m and in the middle of slab when *f* = 90 °C/m. The maximum initial gap height in Fig. 5i when *f* = 90 °C/m is about 1.1 mm, and the value is much larger than that in Fig. 4i when *f* = − 45 °C/m. The initial gaps will greatly influence the track dynamics due to the open-closure clapping action of slab track when a moving train passes the area with initial gaps.

**Figure 6:**
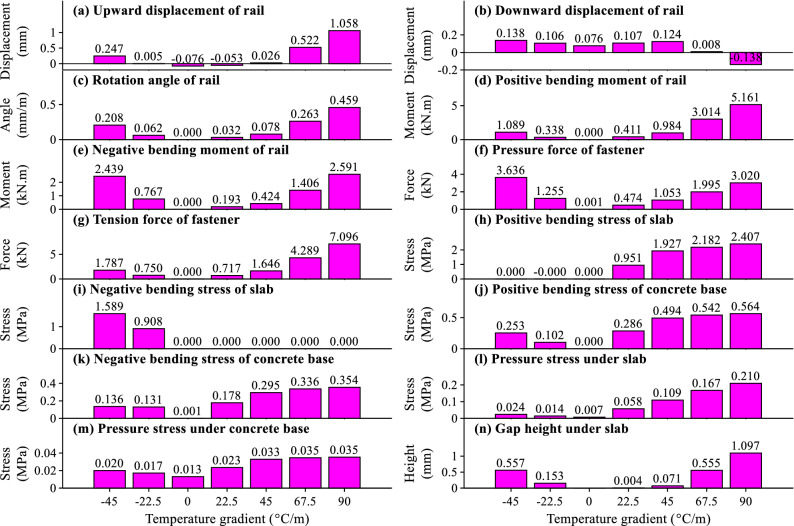
The relation between the temperature gradient of slab track and maximum initial values for different items.

The influences of temperature gradient of slab track on the maximum initial values for different items are plotted in Fig. 6a–n.

It can be concluded from Fig. 6a–n that with the increasing temperature gradient of slab track, most of the maximum initial values for different items will increase. However, the increasing laws for different items are different. The maximum initial upward displacement and rotation angle of rail in Fig. 6a, c, positive and negative rail bending moments in Fig. 6d, e, pressure and tension fastener forces in Fig. 6f, g, gap height under slab in Fig. 6n increase faster and faster. While the maximum initial positive and negative bending stresses of slab and concrete base in Fig. 6h–k, pressure stress under concrete base in Fig. 6m increase slower and slower.

From the results and discussions above, the temperature gradient of slab track affects the initial slab bending stress, fastener force and pressure stress under concrete base significantly. These items will be further analyzed in “Envelope curves of the dynamic responses for typical load cases”, “Acceleration time histories and frequency distributions for typical load cases”, “Influence of temperature gradient of slab track” sections. Moreover, the high-frequency accelerations of track components due to the slab track’s temperature gradient will propagate to the surrounding soil and building and cause environmental vibration. Thus, the accelerations of track components will have a negative effect on the environmental vibration and have acquired more and more attention in the academic and engineering circles and will also be analyzed in “Envelope curves of the dynamic responses for typical load cases”, “Acceleration time histories and frequency distributions for typical load cases”, “Influence of temperature gradient of slab track” sections.

### Envelope curves of the dynamic responses for typical load cases

**Figure 7:**
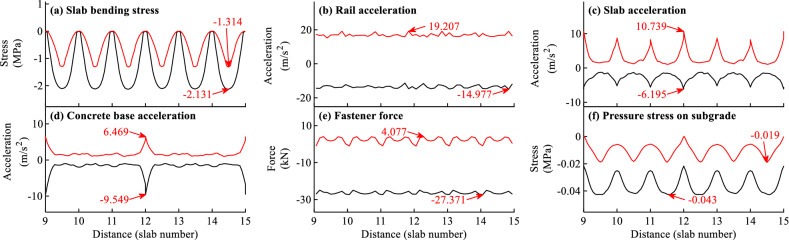
Envelope curves of the dynamic responses for load case when *f* = -45 °C/m.

**Figure 8:**
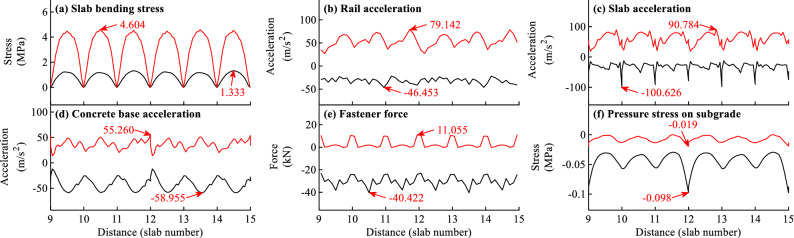
Envelope curves of the dynamic responses for load case when *f* = 90 °C/m.

The envelope curves of the dynamic responses for different calculation items can be obtained by calculating the maximum and minimum value of the time history curve for each node or element along the track. Figures 7a–f and 8a–f show the envelope curves of the dynamic responses for typical load cases when *f* = − 45 and 90 °C/m, respectively.

As shown in Figs. 7–8, the dynamic responses of slab track in Fig. 8 are larger than those in Fig. 7, indicating that the dynamic responses of slab track can be affected by the temperature gradient of slab track.

As shown in Figs. 7–8, the distributions of the dynamic responses are not uniform along the track, and the distribution laws for different items under different temperature gradients of slab track are different. Generally speaking, the distributions of the dynamic responses in Fig. 7 when *f* = − 45 °C/m are more regular than those in Fig. 8 when *f* = 90 °C/m, and the distributions of the slab bending stress, slab acceleration, concrete base acceleration, pressure stress on subgrade are more regular than the rail acceleration and fastener force. It can further be concluded from Figs. 7–8 that the locations where the largest responses are different for different items. Generally, the most unfavorable position for each track component is either at the end or in the middle. For example, the maximum slab bending stresses in Figs. 7a and 8a appear in the slab middle. The maximum slab accelerations in Figs. 7c and 8c appear at the slab end. The maximum concrete base acceleration in Fig. 7d appears at the end of concrete base. The maximum pressure stresses on subgrade in Figs. 7f and 8f respectively appear in the slab middle and at the concrete base end.

By comparing the envelope curves in Figs. 7b–d and 8b–d, it is apparent that the accelerations of different track components are closely related to the temperature gradient load. The dynamic accelerations of different track components when *f* = 90 °C/m are several times larger than those when *f* = − 45 °C/m. For example, the accelerations of rail, slab, concrete base are 19.207, 10.739, 9.549 m/s^2^, respectively when *f* = − 45 °C/m, and are 79.142, 100.626, 58.955 m/s^2^, respectively when *f* = 90 °C/m.

It can be concluded that the temperature gradient load greatly affects the dynamic properties of CRTS III slab track. The conclusion is not consistent with that in Ref.^31^. Two reasons may be attributed to this. On the one hand, the contact nonlinearity of structural interface is not considered in Ref.^31^ while it is considered in this paper. On the other hand, the ballastless track in Ref.^31^ is the CRTS II slab track, which is continuous in longitudinal direction and the temperature deformation is much smaller than the CRTS III slab track in this paper.

### Acceleration time histories and frequency distributions for typical load cases

**Figure 9:**
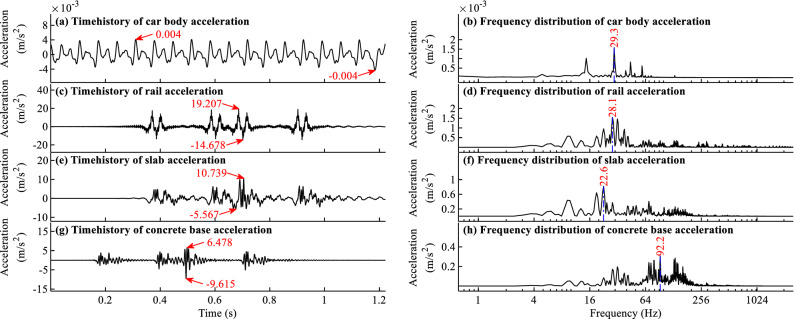
Acceleration time histories and frequency distributions for load case when *f* = − 45 °C/m.

**Figure 10:**
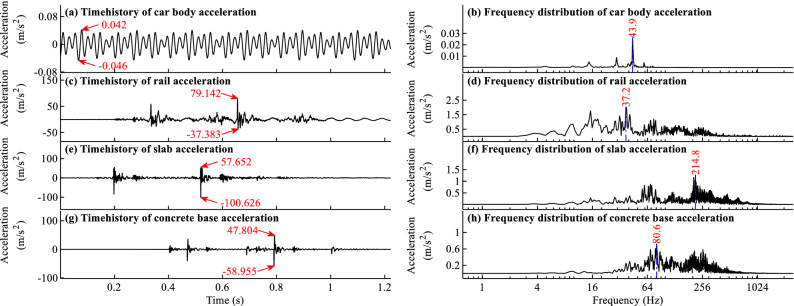
Acceleration time histories and frequency distributions for load case when *f* = 90 °C/m.

The acceleration time histories and frequency distributions of different components with the largest responses for typical load cases when *f* = − 45 °C/m and *f* = 90 °C/m are shown in Figs. 9a–h and 10a–h, respectively.

As shown in Figs. 9a and 10a, the acceleration time history of car body is in regular shape, and the corresponding frequencies for the 4 largest peak points in Figs. 9b and 10b are 14.7, 29.3, 43.9, 58.6 Hz, respectively. The 4 main frequencies of the car body acceleration in Figs. 9b and 10b are about 1, 2, 3, and 4 times as large as the excitation frequency 14.7 Hz, which can be calculated by using the moving train speed 83.333 m/s divide the periodic excitation track irregularities with 5.67 m wavelength shown in Figs. 4a and 5a. The excitation frequency could be well reflected in the main frequencies of car body acceleration, which could verify the simulation results to some extent.

From Figs. 9c, e, g and 10c, e, g, one can find many peaks in the acceleration time history curves, and the peaks appear in the curves when the wheels pass the measuring point. One can further find that the responses are largest when the fifth or sixth wheel passes the measuring point, indicating a large deviation with only one car with 4 wheels considered in the train model. The conclusion is consistent with that in Ref.^33^.

From Figs. 9h and 10f, h, it is evident that many high frequencies above 100 Hz appear in the vibration frequency of slab and concrete base. The reason is that there are gaps under the slab, as shown in Figs. 4i and 5i. When a train passes the gap area, due to the dynamic clapping action of slab track, the vibration is large and the vibration frequency is high. The high-frequency vibration is closely related to the gap, so the nonlinear contact element and time step with a small value are needed in the dynamic simulation to truly reflect the dynamic impact effect in the gap area.

**Figure 11:**
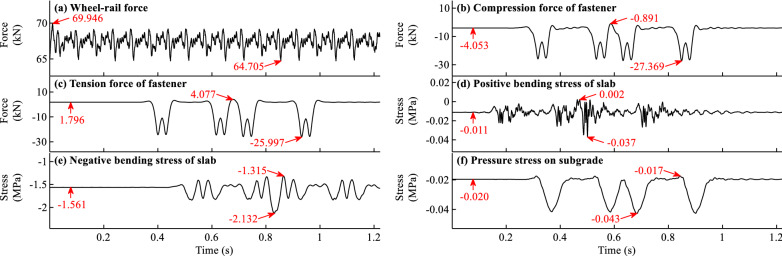
Time histories of the dynamic forces and stresses for load case with *f* = − 45 °C/m.

**Figure 12:**
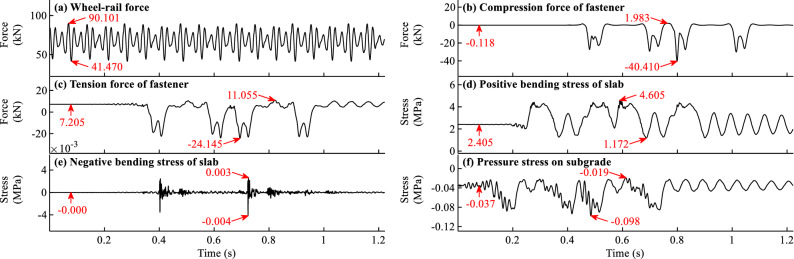
Time histories of the dynamic force and stress for load case with *f* = 90 °C/m.

The time histories of the wheel-rail force, fastener forces, slab bending stresses, pressure stresses on subgrade when *f* = − 45 °C/m and *f* = 90 °C/m are shown in Figs. 11a–f and 12a–f respectively. It should be noted that the time histories in Figs. 11b–f and 12b–f are for the calculation items with the largest responses.

As can be observed in Figs. 11a and 12a, the temperature gradient of slab track has little influence on the wheel-rail force for load case when *f* = − 45 °C/m. However, its influence on the wheel-rail force for load case when* f* = 90 °C/m is significant. The reasons can be attributed to that the initial states of slab track when* f* = − 45 °C/m in Fig. 4a, i and f = 90 °C/m in Fig. 5a, i are different. In Fig. 5a, i, the initial rail displacement and the gap height under slab are much larger than those in Fig. 4a, i.

From Fig. 11b–f and 12b–f, it can be seen that many peaks appear in the time history curves. The peaks in the front and rear part of the curve can be attributed to the action of the first and second cars, respectively. For example, there are 8 peaks in Fig. 12b, and the corresponding time of the first peak indicates that the first wheel of the train passes the measuring point. The response of the fifth peak in Fig. 12b is the largest, indicating that the maximum response occurs when the fifth wheel of the train passes the measuring point. Many peaks with maximum response appear in the rear part of the curve, indicating that there is some deviation with only one car with 4 wheels considered in the train model.

It is apparent from Figs. 11b–f and 12b–f that initial force and stress exist in the time history curves. However, the proportions of initial force and stress in the total responses differ for different calculation items. The initial fastener tension force and bending stress of slab have a large proportion, while the initial fastener pressure force has a small proportion. For example, the initial and maximum fastener tension forces in Fig. 12c are 7.205 and 11.055 kN, respectively, and the initial force accounts for 65.2% of the maximum force. The initial and maximum positive slab bending stresses in Fig. 12d are 2.405 and 4.605 MPa respectively, and the initial stress accounts for 52.2% of the maximum stress. The initial forces and stresses caused by the temperature gradient and gravity load of slab track can significantly influence the dynamic forces and stresses of slab track and cannot be ignored. The coupled effect of the train, temperature gradient, and gravity of slab track should be considered to obtain reasonable dynamic results of slab track structure.

### Influence of temperature gradient of slab track

**Figure 13:**
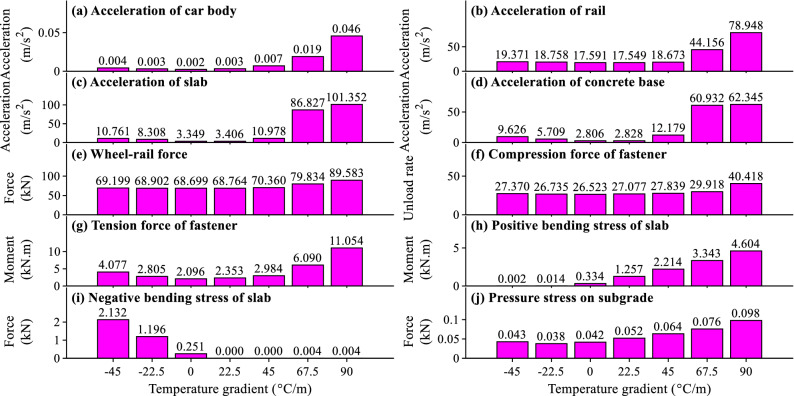
The relation between the temperature gradient of slab track and the maximum dynamic responses.

The influences of temperature gradient of slab track on the maximum dynamic responses for different items of the coupled system are plotted in Fig. 13a–j.

As shown in Fig. 13, the maximum dynamic responses will increase with the increase of the temperature gradient of slab track. However, the increasing laws for different items are different.

As shown in Fig. 13a, the maximum car body acceleration has some relation with the temperature gradient of slab track. However, the maximum car body acceleration 0.046 m/s^2^ in Fig. 13a due to the temperature gradient of slab track is only 3.6% of the allowable car body acceleration 0.13 g^48^ and can be neglected in practical engineering.

As displayed in Fig. 13b–d, the maximum rail, slab, and concrete base accelerations are small when the absolute value of *f* is less than 45 °C/m. They will increase significantly when *f* is larger than 45 °C/m due to the influence of the gap under the slab (see Fig. 5i). As illustrated in Fig. 13b–d, the maximum slab acceleration is larger than the rail and concrete base accelerations when *f* is larger than 45 °C/m. The reason is that the fastener has the vibration-reduction capacity and can reduce the vibration induced by the gap under slab, so the rail acceleration is less than the slab acceleration. The mass of a track slab is much smaller than that of a concrete base, so the concrete base acceleration is also less than the slab. The large vibration of slab track due to large positive temperature gradient load can harm the surrounding environment and should be considered in practical engineering.

It can be observed in Fig. 13e that the temperature gradient of slab track has little influence on the maximum wheel-rail force when the absolute value of *f* is less than 45 °C/m. And the maximum wheel-rail force will increase rapidly when *f* is larger than 45 °C/m. Comparing the maximum wheel-rail force when *f* = 90 °C/m with that when *f* = 45 °C/m, we can deduce that the increasing rate is 27.3%. The maximum wheel-rail force 89.583 kN in Fig. 13e is far less than the allowable wheel-rail force 170 kN^48^, and the running safety of the train can be ensured.

As shown in Fig. 13f, the temperature gradient of slab track has a small and significant influence on the maximum fastener compressive force when *f* is smaller than and larger than 45 °C/m respectively. By comparing the maximum fastener compressive force when *f* = 90 °C/m with that when *f* = 0 °C/m, it can be calculated that the increasing rate is 52.4%. Comparing Fig. 13g with Fig. 13f, the temperature gradient of slab track has a larger influence on the maximum fastener tension force than the compressive force. By comparing the maximum fastener tension force when *f* = 90 °C/m with that when *f* = 0 °C/m, it can be calculated that the increasing rate is 427.4%. The maximum fastener tension force in Fig. 13g is 11.054 kN and is about 61.4% of the allowable fastener tension force 18 kN for WJ-8 fastener system^46^, and the fastener may be damaged under the long-term fatigue load.

As apparent in Fig. 13h–i, the temperature gradient of slab track has a huge influence on the maximum slab bending stresses. The increasing rates for the maximum positive and negative slab bending stresses are 12.79, 7.50 times, respectively. Further analyses show that the maximum slab bending stress in Fig. 13h exceeds the concrete tension stress 2.85 MPa for C60 grade concrete in the design code^47^, and prestress technology and reinforcing bars should be used to improve the durability of slab.

As apparent in Fig. 13j, the temperature gradient of slab track has a large influence on the maximum pressure stress on subgrade. By comparing the maximum pressure stress on subgrade when *f* = 90 °C/m with that when *f* = 0 °C/m, it can be deduced that the increasing rate is 1.33 times. It can also be deduced that the maximum pressure stress on subgrade in Fig. 13j is about 54.4% of the allowable pressure stress on subgrade^49^ and should be considered in practical engineering.

## Conclusions

In this paper, considering the contact nonlinear, the influences of temperature gradient of slab track on the dynamic characteristics of the coupled system are theoretically studied using a coupled nonlinear dynamic model. The following conclusions are drawn.

1. The increasing laws of the maximum initial value for different items of CRTS III slab track on subgrade are different. With the increase of the temperature gradient of slab track, the maximum initial upward displacement and rotation angle of rail, pressure and tension fastener forces, gap height under slab increase faster and faster. While the maximum initial positive and negative bending stresses of slab and concrete, pressure stress under concrete base increase slower and slower.
2. The proportions of initial force and stress in the total dynamic responses differ for different calculation items. The fastener tension force and positive slab bending stress have large proportions exceeding 50%. Therefore, the coupled effect of the moving train, temperature gradient of slab track, and gravity of slab track should be considered.
3. There will appear gaps in the slab track when the temperature gradient of slab track is large. There are many high frequencies above 100 Hz in the vibration frequency of track components, and the nonlinear contact elements are needed in the simulation model to truly reflect the high-frequency dynamic open and closure clapping action between the track components.
4. The distributions of the maximum track dynamic responses are not uniform along the track. Generally, the most unfavorable position for each track component is either at the end or in the middle. The maximum slab bending stress, slab acceleration, concrete base acceleration appear in the slab middle, at the slab end, and at the concrete base end, respectively.
5. The maximum accelerations of track components appear when the fifth or sixth wheel passes the measuring point. There is a large deviation with only one car with 4 wheels considered in the train model, and at least two cars should be used in the train model.
6. The temperature gradient of slab track has different influence laws on the maximum system dynamic responses for different items. It has a small influence on the maximum car body acceleration. However, the influences on the slab acceleration, concrete base acceleration, fastener tension force are large, and the influence on the slab bending stress is huge.

## Data Availability

The datasets used and analysed during the current study are available from the corresponding author on reasonable request.

## References

[1] Gautier PE (2015). Slab track: Review of existing systems and optimization potentials including very high speed. Constr. Build. Mater..

[2] Yuan XC, Tian GY, Wang KY, Zhai WM (2016). Analysis on the dynamic performance of a high-speed train running on different types of ballastless track structures. Int. Conf. Transp. Dev..

[3] Zeng XH (2018). Deterioration mechanism of CA mortar due to simulated acid rain. Constr. Build. Mater..

[4] Liu XC, Yu ZW, Xiang P, Jin C (2019). Composite action of the track slab and the self-compacting concrete filling layer subjected to train-induced fatigue load: An experimental investigation. Proc. Inst. Mech. Eng. Part F J Rail Rapid Transit..

[5] Ou ZM, Li FJ (2014). Analysis and prediction of the temperature field based on in-situ measured temperature for CRTS-II ballastless track. Energy Procedia..

[6] Yang RS, Li JL, Kang WX, Liu XY, Cao SH (2017). Temperature characteristics analysis of the ballastless track under continuous hot weather. J. Transp. Eng. Part A Syst..

[7] Lou P, Zhu JP, Dai GL, Yan B (2018). Experimental study on bridge-track system temperature actions for Chinese high-speed railway. Arch. Civ. Mech. Eng..

[8] Liu S, Chen XH, Yang J, Cai DG, Yang GT (2020). Numerical study and in-situ measurement of temperature features of asphalt supporting layer in slab track system. Constr. Build. Mater..

[9] Zhao L (2020). Experimental study of the temperature distribution in CRTS-II ballastless tracks on a high-speed railway bridge. Appl. Sci..

[10] Jiang HL, Zhang JW, Zhou F, Wang Y (2020). Optimization of PCM coating and its influence on the temperature field of CRTS II ballastless track slab. Constr. Build. Mater..

[11] Ren JJ, Deng SJ, Jin ZB, Yang JB (2017). Energy method solution for the vertical deformation of longitudinally coupled prefabricated slab track. Math. Probl. Eng..

[12] Chen Z, Xiao JL, Liu XK, Liu XY (2018). Effects of initial up-warp deformation on the stability of the CRTS II slab track at high temperatures. J. Zhejiang Univ. SCI A.

[13] Chen Z, Xiao JL, Liu XY, Qin HP, Yang RS (2019). Deformation behavior of slab warping for longitudinal continuous rigid slab under temperature effect. Adv. Struct. Eng..

[14] Cai XP, Luo BC, Zhong YL, Zhang YR, Hou BW (2019). Arching mechanism of the slab joints in CRTS II slab track under high temperature conditions. Eng. Fail. Anal..

[15] Cho YK, Kim SM, Chung W, Kim JC, Oh HJ (2014). Effect of steel ratio on behavior of continuously reinforced concrete railway track under environmental loads. KSCE J. Civ. Eng..

[16] Liu XK, Zhang WH, Xiao JL, Liu XY, Li W (2019). Damage mechanism of broad-narrow joint of CRTS II slab track under temperature rise. KSCE J. Civ. Eng..

[17] Li Y, Chen JJ, Wang JX, Shi XF, Chen L (2020). Study on the interface damage of CRTS II slab track under temperature load. Structures.

[18] Li Y, Chen JJ, Wang JX, Shi XF, Wang R (2021). Interfacial failure and arching of the CRTS II slab track reinforced by post-installed reinforcement bars due to thermal effects. Eng. Fail. Anal..

[19] Xu YD, Yan DB, Zhu WJ, Zhou Y (2020). Study on the mechanical performance and interface damage of CRTS II slab track with debonding repairment. Constr. Build. Mater..

[20] Cui XH (2021). Interface damage and arching mechanism of CRTS II slab track under temperature load. Constr. Build. Mater..

[21] Zhong YL, Gao L, Zhang YR (2018). Effect of daily changing temperature on the curling behavior and interface stress of slab track in construction stage. Constr. Build. Mater..

[22] Zhong YL (2021). An improved cohesive zone model for interface mixed-mode fractures of railway slab tracks. Appl. Sci..

[23] Zhu SY (2018). Mechanical property and damage evolution of concrete interface of ballastless track in high-speed railway: Experiment and simulation. Constr. Build. Mater..

[24] Song L, Liu HB, Cui CX, Yu ZW, Li ZG (2020). Thermal deformation and interfacial separation of a CRTS II slab ballastless track multilayer structure used in high-speed railways based on meteorological data. Constr. Build. Mater..

[25] Xu QY, Li B (2012). Study on spatial mechanical characteristic of high-speed railway ballastless slab track on subgrade. Adv. Mater. Res..

[26] Ren JJ, Li X, Yang RS, Wang P, Xie P (2016). Criteria for repairing damages of CA mortar for prefabricated framework-type slab track. Constr. Build. Mater..

[27] Zhang YR, Wu K, Gao L, Yan S, Cai XP (2019). Study on the interlayer debonding and its effects on the mechanical properties of CRTS II slab track based on viscoelastic theory. Constr. Build. Mater..

[28] Wang JF, Zhou YB, Wu TM, Wu X (2019). Performance of cement asphalt mortar in ballastless slab track over high-speed railway under extreme climate conditions. Int. J. Geomech..

[29] Li Y, Chen JJ, Wang JX, Shi XF, Chen L (2020). Analysis of damage of joints in CRTS II slab track under temperature and vehicle Loads. KSCE J. Civ. Eng..

[30] Zhu SY, Cai CB (2014). Interface damage and its effect on vibrations of slab track under temperature and vehicle dynamic loads. Int. J. Non Linear Mech..

[31] Song XL, Zhao CF, Zhu XJ (2014). Temperature-induced deformation of CRTS II slab track and its effect on track dynamical properties. Sci. China Technol. Sci..

[32] Gao L, Zhao WQ, Hou B, Zhong YL (2020). Analysis of influencing mechanism of subgrade frost heave on vehicle-track dynamic system. Appl. Sci..

[33] Xu QY (2021). Influence of vehicle number on the dynamic characteristics of high-speed train- CRTS III slab track-subgrade coupled system. Materials.

[34] Lou P, Gong KL, Zhao C, Xu QY, Luo RK (2019). Dynamic responses of vehicle-CRTS III slab track system and vehicle running safety subjected to uniform seismic excitation. Shock Vib..

[35] Xu L, Yu ZW (2021). Dynamic solution for vehicle-track interaction considering the elastoplasticity of track slabs. J. Vib. Control..

[36] Xu L, Xin LF, Yu ZW, Zhu ZH (2020). Construction of a dynamic model for the interaction between the versatile tracks and a vehicle. Eng. Struct..

[37] Xu QY, Yan B, Lou P, Zhou XL (2015). Influence of slab length on dynamic characteristics of subway train-steel spring floating slab track-tunnel coupled system. Lat. Am. J. Solids Struct..

[38] Nguyen K, Goicolea JM, Galbadon F (2014). Comparison of dynamic effects of high-speed traffic load on ballasted track using a simplified two-dimensional and full three-dimensional model. Proc. Inst. Mech. Eng. Part F J Rail Rapid Transit..

[39] Zhai W, Cai Z (1997). Dynamic interaction between a lumped mass vehicle and a discretely supported continuous rail track. Comput. Struct..

[40] Lei XY, Noda NA (2002). Analyses of dynamic response of vehicle and track coupled system with random irregularity of track vertical profile. J. Sound Vibr..

[41] Xu QY, Chen XP, Yan B, Guo W (2015). Study on vibration reduction slab track and adjacent transition section in high-speed railway tunnel. J. Vibroeng..

[42] Chen ZW, Zhai WM, Yin Q (2018). Analysis of structural stresses of tracks and vehicle dynamic responses in train-track-bridge system with pier settlement. Proc. Inst. Mech. Eng. Part F J Rail Rapid Transit..

[43] Zhu SY, Yang JZ, Cai CB (2017). Application of dynamic vibration absorbers in designing a vibration isolation track at low-frequency domain. Proc. Inst. Mech. Eng. Part F J Rail Rapid Transit..

[44] Chinese Railway Corporation. Code for design of railway track (Limit state method) (QCR 9130-2018). China Railway Publishing House (2018) (in Chinese).

[45] Ministry of Railways of the People’s Republic of China. Code for design of railway continuous welded rail (TB 10015-2012). China Railway Publishing House (2013) (in Chinese).

[46] National Railway Administration of People’s Republic of China. Code for design of railway track (TB 10082-2017). China Railway Publishing House (2017) (in Chinese).

[47] Ministry of Housing and Urban-Rural Construction of the People’s Republic of China. Code for design of concrete structures (GB 50010-2010). China Architecture & Building Press (2010) (in Chinese).

[48] National Railway Administration of People’s Republic of China. Code for design of high-speed railway (TB 10621-2014). China Railway Publishing House (2014) (in Chinese).

[49] Ren JJ, Yang RS, Wang P, Dai F, Yan XB (2017). Influence of contact loss underneath concrete underlayer on dynamic performance of prefabricated concrete slab track. Proc. Inst. Mech. Eng. Part F J Rail Rapid Transit..

